# Human RIF1-Protein Phosphatase 1 Prevents Degradation and Breakage of Nascent DNA on Replication Stalling

**DOI:** 10.1016/j.celrep.2019.05.002

**Published:** 2019-05-28

**Authors:** Javier Garzón, Sebastian Ursich, Massimo Lopes, Shin-ichiro Hiraga, Anne D. Donaldson

**Affiliations:** 1 Institute of Medical Sciences, University of Aberdeen, Foresterhill, Aberdeen AB25 2ZD, UK; 2 Institute of Molecular Cancer Research, University of Zürich, 8057 Zürich, Switzerland

**Keywords:** DNA replication, fork protection, replication stress, genome integrity, RIF1-PP1, DNA2, WRN

## Abstract

RIF1 is a multifunctional protein implicated in controlling DNA replication and repair. Here, we show that human RIF1 protects nascent DNA from over-degradation at stalled replication forks. The major nuclease resecting nascent DNA in the absence of RIF1 is DNA2, operating with WRN as an accessory helicase. We show that RIF1 acts with protein phosphatase 1 to prevent over-degradation and that RIF1 limits phosphorylation of WRN at sites implicated in resection control. Protection by RIF1 against inappropriate degradation prevents accumulation of DNA breakage. Our observations uncover a crucial function of human RIF1 in preventing genome instability by protecting forks from unscheduled DNA2-WRN-mediated degradation.

## Highlights

• Human RIF1 protects nascent DNA at stalled replication forks
• PP1 interaction is essential for RIF1 to prevent degradation upon fork stalling
• DNA2 nuclease together with WRN helicase mediates resection in the absence of RIF1
• DNA2-mediated degradation in cells lacking RIF1 leads to DNA damage

## Graphical Abstract

**Figure:**
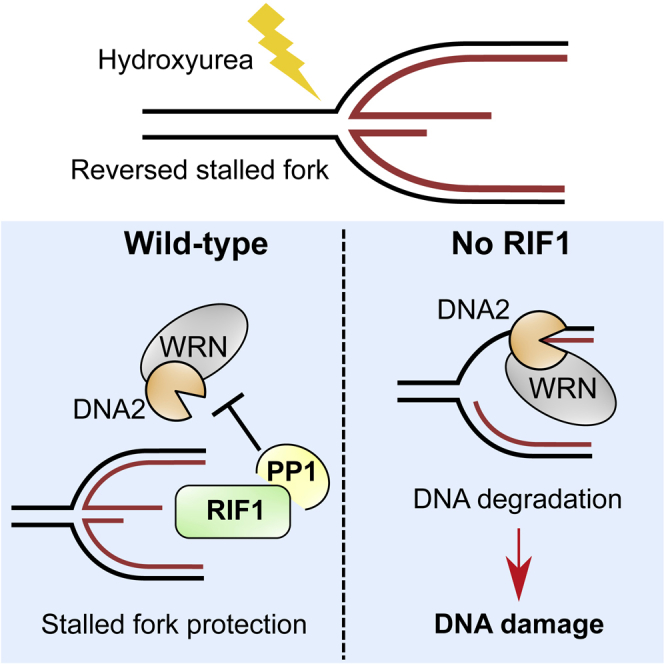

## Teaser

Garzón et al. show that human RIF1 protects nascent DNA from DNA2-WRN-mediated degradation upon replication fork stalling. This function depends on RIF1 interaction with PP1 and prevents the accumulation of DNA damage and breaks. This discovery highlights the importance of RIF1 in maintaining genomic stability when DNA replication is challenged.

## Introduction

Accurate DNA replication is essential to maintain genomic stability through successive cell cycles. Genome duplication is, however, challenged by threats such as DNA lesions, collisions with the transcription machinery, and nucleotide pool depletion (Zeman and Cimprich, 2014). Preserving the integrity of stalled replication forks is essential to prevent genome instability-inducing events that may cause oncogenesis. Cells rely on various mechanisms to protect against replication-related damage, including S phase checkpoint activation, which stabilizes protein complexes at stalled forks and suppresses late origin firing to delay cell-cycle progression (Smith et al., 2010).

An additional layer of control emerged with the understanding that the tumor suppressor protein BRCA2, traditionally associated with homologous recombination repair, also protects nascent DNA at stalled replication forks (Schlacher et al., 2011). Specifically, upon treatment with the replication inhibitor hydroxyurea (HU), BRCA2-defective cells showed increased degradation of nascent DNA strands. This resection is due to MRE11 nuclease and is caused by the inability of BRCA2-deficient cells to stabilize RAD51 nucleofilaments at blocked forks (Schlacher et al., 2011, Schlacher et al., 2012). Recent studies have revealed the molecular mechanisms underlying resection in BRCA1- and BRCA2-defective cells, by demonstrating that reversed replication forks act as the entry point for the nucleolytic degradation (Lemaçon et al., 2017, Mijic et al., 2017, Taglialatela et al., 2017). Various nucleolytic pathways appear to be differentially regulated by distinct fork protective proteins. The DNA2 nuclease promotes nucleolytic degradation after prolonged HU treatment in wild-type U2OS cells (Thangavel et al., 2015), although DNA2 is not the major nuclease responsible for resection in the BRCA mutants (Lemaçon et al., 2017). Factors that limit DNA2-mediated processing include the Abraxas paralog Abro1, BOD1L, and CtIP (Higgs et al., 2015, Przetocka et al., 2018, Xu et al., 2017a). Unscheduled DNA2-dependent degradation of newly replicated DNA in the absence of these proteins causes catastrophic genome instability upon replication fork stalling. The importance of the nascent DNA protection pathways was underscored by a study identifying the acquisition of nascent DNA protection as a new mechanism for the development of chemoresistance in BRCA-deficient tumor cells (Ray Chaudhuri et al., 2016).

RIF1 has emerged as a multifunctional chromosome stability protein playing diverse roles in mammalian cells. In double-strand break (DSB) repair, mammalian RIF1 acts with 53BP1 to promote non-homologous end joining by antagonizing BRCA1-mediated processing of the break ends (Chapman et al., 2013, Escribano-Díaz et al., 2013). RIF1 also negatively regulates DNA initiation at replication origins in both yeast and human cells by acting as a protein phosphatase 1 (PP1) substrate-targeting subunit (Hiraga et al., 2014, Hiraga et al., 2017), counteracting the Dbf4-dependent kinase (DDK)-dependent phosphorylation that activates the minichromosome maintenance protein (MCM) complex as the major replicative helicase. Interaction of PP1 with its substrate-targeting subunits is mediated through characteristic amino acid motifs (RVxF and SILK sequences). Human RIF1 contains three such PP1-interacting motifs, which mediate its association with PP1 and are required for RIF1 to negatively regulate replication initiation (Hiraga et al., 2017).

RIF1-deficient cells are hypersensitive to several replication inhibitors (Buonomo et al., 2009, Xu et al., 2017b), and human RIF1 localizes to replication forks independently of 53BP1 (Alabert et al., 2014). However, the specific role of RIF1 at stalled forks and its response to replication inhibition has remained largely unclear. Here, we show that through its interaction with PP1, human RIF1 prevents the untimely degradation of newly replicated DNA at replication forks challenged with HU. Moreover, we identify DNA2, acting with the Werner’s helicase protein WRN, as the major nuclease-helicase complex that drives the uncontrolled resection of nascent DNA in the absence of RIF1. We find that RIF1 regulates WRN helicase phosphorylation at residues implicated in the control of resection. We also reveal that nascent strand protection by RIF1 is essential to prevent the accumulation of DNA damage in HU-treated cells.

## Results

### RIF1 Protects Nascent DNA from Degradation upon Fork Stalling through Its Interaction with Protein Phosphatase 1

**Figure 1:**
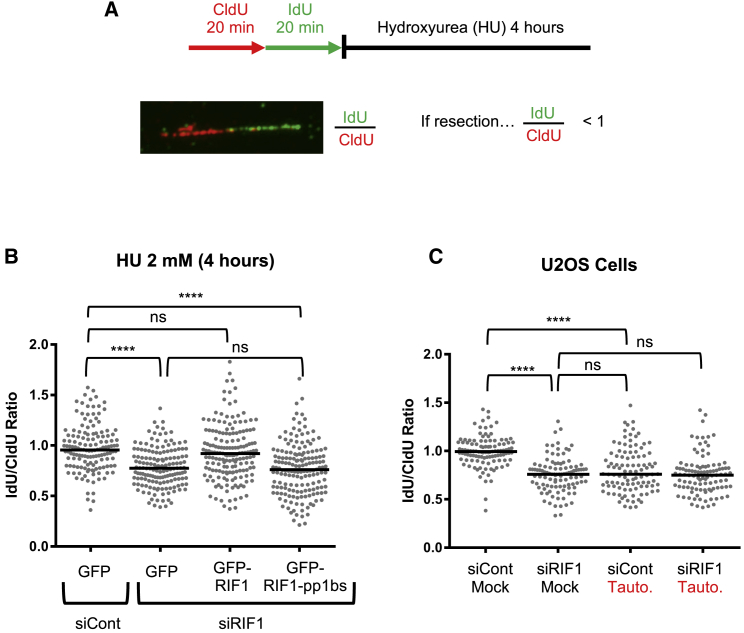
RIF1 Prevents DNA Resection at Stalled Forks through PP1 Interaction (A) Schematic diagram of fork protection assay. (B) Nascent DNA degradation analysis in HEK293-derived cell lines transfected with siControl or siRIF1 and expressing constructs indicated. Cells were treated with HU 2 mM for 4 h before fiber analysis. For fiber analysis through the whole study, at least 100 forks were evaluated per condition. The bar reflects the median value, and the statistical analysis was performed using the Mann-Whitney test. (C) Resection of nascent DNA in U2OS cells, evaluated as in (B). Cells were mock treated or treated with tautomycetin at 5 μM, as indicated. ns, not significant; ^∗∗∗∗^p ≤ 0.0001.

RIF1-deficient cells have been reported to show hypersensitivity to some replication inhibitors, highlighting the importance of RIF1 in replication stress survival. These observations prompted us to investigate any role for human RIF1 in controlling nascent DNA stability upon replication blockage. We first performed DNA fiber analysis to test whether the absence of RIF1 leads to unscheduled resection of the newly replicated DNA. Cells were treated successively with the thymidine analogs 5-chloro-2ʹ-deoxyuridine (CldU) and 5-iodo-2ʹ-deoxyuridine (IdU), followed by HU treatment (Figure 1A). Immunodetection-based visualization of the IdU- and CldU-labeled tracts on DNA fibers then allows the extent of the degradation of nascent DNA to be monitored by assessing the IdU:CldU length ratio. To test RIF1 function at stalled forks, we used previously established HEK293-derived cell lines (Hiraga et al., 2017), which contain doxycycline (DOX)-inducible cDNA constructs encoding GFP-fused RIF1, or GFP as a control. Depletion of endogenous RIF1 from the control cell line caused significantly increased resection of the IdU tract when compared to small interfering control RNA (siControl)-transfected cells (Figure 1B, columns 1 and 2, GFP samples), suggesting a role for RIF1 in protecting nascent DNA upon fork stalling. DOX-induced expression of GFP-RIF1 prevented the degradation of nascent DNA in this endogenous RIF1-depleted background, confirming the specificity of siRIF1 treatment and the functionality of the GFP-RIF1 protein (Figure 1B, GFP-RIF1).

Since several roles of RIF1 require its interaction with PP1 (Hiraga et al., 2014, Hiraga et al., 2017, Kedziora et al., 2018), we tested whether PP1 may mediate this effect of RIF1 at stalled forks. For this purpose, we used a cell line containing a DOX-inducible construct encoding a GFP-fused mutant RIF1 (GFP-RIF1-pp1bs) that has all three PP1-interaction motifs mutated so that it can no longer bind PP1 (Hiraga et al., 2017). The GFP-RIF1 and GFP-RIF1-pp1bs constructs are resistant to siRNA knockdown due to synonymous base substitutions. The depletion of endogenous RIF1, followed by the induction of GFP-RIF1-pp1bs failed to restore the IdU:CldU ratio (Figure 1B, GFP-RIF1-pp1bs), in contrast to GFP-RIF1. The association of PP1 with RIF1 is therefore required to preserve the integrity of nascent DNA at blocked forks. In unperturbed conditions, the IdU:CldU ratio was unaffected by RIF1 knockdown or by the expression of GFP-fused constructs (Figures S1A and S1B), confirming that these effects reflect events that occur after fork blocking and not during normal replication. CldU tract length was slightly increased in RIF1-depleted cells before HU addition when compared to siControl (Figure S1C), suggesting a slightly (26%) increased progression rate of unperturbed forks in the absence of RIF1. The difference is statistically significant in this study, probably due to the higher number of forks analyzed here when compared to previous investigations (Chapman et al., 2013, Hiraga et al., 2017) and will be described further elsewhere.

We tested whether the role of RIF1 in nascent DNA fork protection was conserved in another human cell line. In U2OS cells, RIF1 depletion also caused a reduced IdU:CldU ratio after treatment with HU (Figure 1C). Moreover, the chemical inhibition of PP1 using tautomycetin caused nascent DNA resection, mimicking the phenotype of RIF1-depleted cells. Notably, PP1 inhibition did not cause a further increase in degradation in siRIF1-treated cells, implying that RIF1 and PP1 act in the same pathway to protect newly replicated DNA. We also tested replication fork restart capacity in the absence of RIF1, using a fiber assay that monitors the proportion of forks able to resume replication, as assessed by their successful incorporation of IdU after the removal of HU (Figure S1D). However, after treatment with HU, we found no significant decrease in the percentage of forks that were able to restart in the absence of RIF1 (Figure S1D, left panel) or difference in IdU tract length (Figure S1D, right panel).

These results demonstrate that RIF1, through its association with PP1, protects nascent DNA from degradation upon fork stalling due to HU. RIF1 is, however, dispensable for the resumption of DNA synthesis.

### RIF1-Depleted Cells Accumulate ssDNA, Hyperactivate the S Phase Checkpoint, and Show Hypersensitivity to HU Treatment

**Figure 2:**
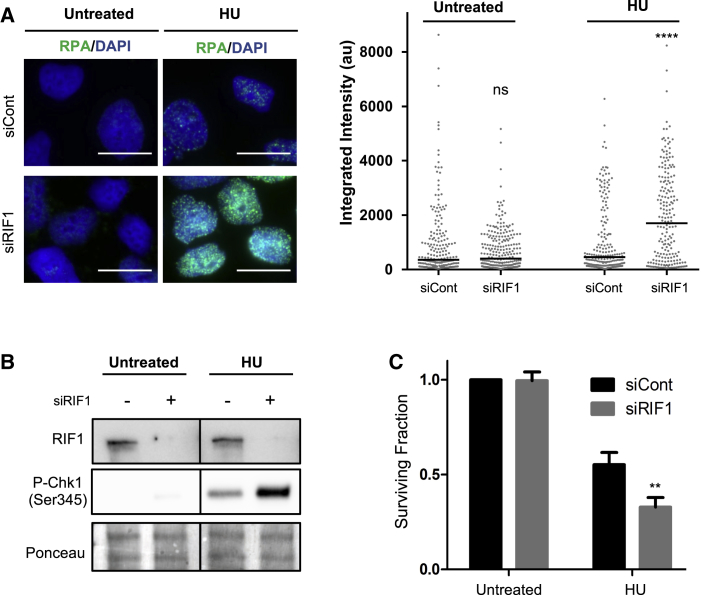
RIF1 Depletion Leads to Increased ssDNA Regions, Hyperactivation of S Phase Checkpoint, and Increased Sensitivity to HU (A) RPA nuclear foci analyzed by immunofluorescence in siControl or siRIF1-treated HEK293 cells. HU treatment was 2 mM for 4 h. Cells were pre-extracted to detect only chromatin-bound RPA. Left, representative images (scale bars, 10 μm). Right, quantification of RPA integrated intensity per nucleus. (B) Whole-cell extracts from HEK293 cells transfected with siControl or siRIF1 analyzed by immunoblotting with the indicated antibodies. HU treatment was 2 mM for 4 h. (C) Clonogenic assay. HEK293 cells were transfected with siControl or siRIF1, treated (or not) with 4 mM HU for 8 h, then cell viability was assessed by counting surviving colonies 7 days after treatment (n = 3 biological replicates). Error bars represent SD. ns, not significant; ^∗∗^p < 0.01; ^∗∗∗∗^p ≤ 0.0001.

Abnormal resection of nascent DNA occurring at stalled forks in the absence of RIF1 could lead to longer stretches of single-stranded DNA (ssDNA), so we examined replication protein A (RPA) foci as a readout for ssDNA accumulation. Staining RIF1-depleted cells revealed an increase in the integrated RPA signal per nucleus compared to controls (Figure 2A). We also observed hyperphosphorylation of Chk1-Ser345 upon HU treatment of cells lacking RIF1 (Figure 2B), reflecting hyperactivation of the S phase checkpoint under replication stress conditions when RIF1 is not present. These data suggest that unscheduled degradation in the absence of RIF1 contributes to the generation of extended ssDNA regions, leading to increased activation of the S phase checkpoint. Moreover, we observed a reduced viability of RIF1-depleted cells treated with HU (Figure 2C), as previously reported.

These results highlight the crucial role of RIF1 in preventing replication stress-associated damage and limiting the activation of the ATR-Chk1 DNA damage checkpoint pathway.

### DNA2 Acts in Conjunction with WRN Helicase to Promote Resection of Stalled Forks in the Absence of RIF1

**Figure 3:**
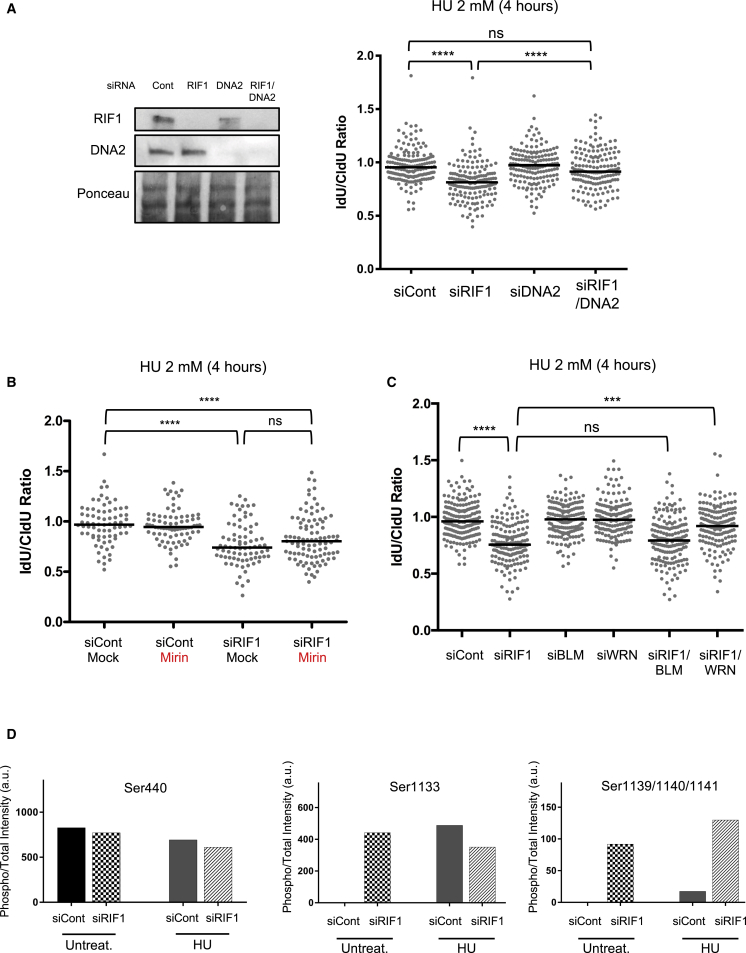
DNA2 and WRN Mediate Nascent DNA Degradation in RIF1-Depleted Cells (A) HEK293 cells were depleted for RIF1 and/or DNA2, and nascent DNA degradation was analyzed after HU treatment (2 mM, 4 h). Left, whole-cell extracts analyzed by immunoblotting, with the indicated antibodies after siRNA transfection. Right, IdU:CldU ratios. (B) Fork protection assay after HU-induced fork stalling in control and RIF1-depleted cells, either mock treated or treated with 50 μM mirin to inhibit MRE11 nuclease activity. (C) Nascent DNA degradation after RIF1, BLM, and/or WRN knockdown in HEK293 cells. Cells were treated with HU and degradation analyzed as in (A) and (B). (D) Analysis of WRN phosphorylation in RIF1-depleted cells. FLAG-WRN was overexpressed in HEK293 cells transfected with siRIF1 or siControl. Cells were collected in unperturbed conditions or after treatment with HU 2 mM for 4 h. FLAG-WRN was immunoprecipitated and analyzed by mass spectrometry (MS). Bar charts show the intensity of peptides with the indicated phosphorylation sites. MS1 values for each site were normalized by the summed intensities of total WRN in each sample. HU sample values correspond to the average of two independent biological replicates. ns, not significant; ^∗∗∗^p < 0.001; ^∗∗∗∗^p ≤ 0.0001.

We sought to identify the nucleases responsible for the increased degradation when RIF1 is not present. Co-depletion of DNA2 and RIF1 considerably ameliorated the nascent DNA degradation seen in cells lacking RIF1 alone upon fork stalling (Figure 3A). Treatment with a chemical inhibitor of DNA2, NSC-105808, similarly suppressed overresection in cells depleted of RIF1 (Figure S2A). In U2OS cells, simultaneous removal of DNA2 and RIF1 also protected nascent DNA from degradation, demonstrating that this resection pathway acts similarly in another human cell line (Figure S2B). Overall, these results implicate DNA2 as important for mediating resection in the absence of RIF1.

We examined whether other nucleases contribute to resection in RIF1-depleted cells. MRE11 is the major nuclease promoting degradation at stalled forks in BRCA1, BRCA2, or FANCD2-defective cells (Schlacher et al., 2011, Schlacher et al., 2012). However, inhibition of MRE11 with mirin (Figure 3B) or its depletion by siMRE11 (Figure S2C) caused only a slight reduction in nascent DNA degradation in RIF1-deficient cells, with the minor effects observed not being statistically significant. Nor did EXO1 depletion significantly affect the extent of DNA degradation in cells lacking RIF1 (Figure S2D). Overall, these data strongly suggest that RIF1 suppresses specifically DNA2-mediated degradation of newly replicated DNA at stalled forks, working independently from the BRCA2-dependent pathway of fork protection. Simultaneous BRCA2 depletion increased nascent DNA degradation compared to RIF1 depletion alone, consistent with the suggestion that RIF1 and BRCA2 operate in separate pathways to suppress resection (Figure S2E).

RIF1 is not the first factor that acts in fork protection by suppressing DNA2-mediated degradation. Abro1 and CtIP, for example, were recently found to limit nucleolytic processing by preventing DNA2-mediated degradation (Przetocka et al., 2018, Xu et al., 2017a); co-depletion of CtIP with RIF1 did not cause increased degradation (Figure S2E), suggesting that CtIP opposes the same degradation pathway as RIF1.

DNA2 nuclease has been reported to cooperate with either Bloom’s (BLM) or Werner’s (WRN) helicase to promote DNA end resection (Nimonkar et al., 2011, Sturzenegger et al., 2014), so we evaluated the contribution of these helicases. While BLM depletion did not significantly improve nascent DNA protection in cells lacking RIF1, co-depletion of WRN with RIF1 largely prevented the extensive degradation (Figures 3C and S3A), implying that WRN is crucial for the resection of stalled forks in the absence of RIF1 and suggesting that this helicase collaborates with DNA2 nuclease in resecting the unprotected nascent DNA. *In vitro* studies have shown that human WRN interacts with DNA2 to stimulate the resection of 5′-recessed DNA ends and degradation of double-stranded DNA (dsDNA) (Pinto et al., 2016, Sturzenegger et al., 2014). A previous study indicated that stalled forks must be reversed to form the substrate for DNA2 nuclease (Thangavel et al., 2015), leading us to investigate the importance of fork reversal for degradation. Upon fork stalling, RAD51 is needed for fork reversal to occur (Zellweger et al., 2015). We found that RAD51 depletion from cells lacking RIF1 suppressed the resection of the nascent DNA (Figure S4A). Depletion of SMARCAL1, a translocase that is also required for fork reversal (Bétous et al., 2012), similarly suppressed degradation (Figure S4B). These effects strongly suggest that reversed forks generated upon HU treatment are the main substrate for degradation by DNA2-WRN when RIF1 is not present.

### WRN Is Hyperphosphorylated in the Absence of RIF1

Our DNA fiber experiments implicate PP1 as acting with RIF1 to prevent nascent DNA degradation, suggesting that protection requires the dephosphorylation of factor(s) at stalled forks. As the factors that mediate resection, both DNA2 nuclease and WRN helicase are potential candidates for dephosphorylation by RIF1-PP1. DNA2 phosphorylation status has not been reported to affect its resection activity in higher eukaryotes. Some evidence is available concerning phosphorylation-mediated regulation of human WRN. In particular, it was reported that cyclin-dependent kinase 1 (CDK1) phosphorylates WRN (at S1133) to control DNA2-dependent end resection at DSBs arising during replication (Palermo et al., 2016). This study, together with our observation of increased WRN phosphorylation in RIF1-deficient cells in a preliminary phosphoproteomic analysis (Hiraga et al., 2017; data not shown), prompted us to examine WRN as a possible target of RIF1-PP1. Substrates dephosphorylated by RIF1-PP1 are expected to show increased phosphorylation when RIF1 is absent. To identify residues hyperphosphorylated under RIF1-deficient replication-blocked conditions, we performed immunoprecipitation of overexpressed FLAG-tagged WRN from siRIF1 or siControl cells, either in unperturbed cells or after HU treatment (Figure S3B). Label-free mass spectrometry analysis identified 12 quantifiable phosphorylation sites within the WRN sequence. While most sites were not affected by RIF1 loss (e.g., Serine 440, Figure 3D, left panel), we identified a cluster of residues in which RIF1 depletion did affect phosphorylation levels either without or with HU treatment. Phosphorylation of the S1133 residue was undetectable in the siControl untreated cells, but it was prominently observed in RIF1-depleted untreated cells (Figure 3D, center panel). S1133 phosphorylation was also high following HU treatment, irrespective of the presence of RIF1. Moreover, we identified phosphorylation in a group of three serines at positions 1139–1141 (sequence 1139-SSSQPV-1144) that showed greatly increased intensity in the RIF1-deficient samples, compared to siControl, in both untreated and HU-treated conditions (Figure 3D, right panel). CDK-mediated S1133 phosphorylation was previously shown to promote resection by DNA2-WRN (Palermo et al., 2016), and phosphorylation of the S1141 residue appears to modulate WRN activity (Su et al., 2016). Both of these studies focused on the role of WRN in the context of DSBs arising after camptothecin treatment. Our observations raised the suggestion that upon HU blockage, phosphorylation of the WRN S1133 site and the S1139/40/41 cluster may contribute to the aberrant overresection observed in RIF1-depleted cells. We examined the impact of mutating these residues in an experiment in which we depleted endogenous WRN and instead expressed a mutant 4A WRN protein replacing serine residues 1133, 1139, 1140, and 1141 with alanine. We found that the mutant protein was still able to promote nascent DNA degradation (Figure S3C). This result implies that while they may contribute, phosphorylation of these four residues is dispensable for degradation. It seems likely therefore that other phosphosites exist, not identified by our experiments, that are also regulated by RIF1 and important for the control of WRN helicase.

The MCM complex is well characterized as a target for dephosphorylation by RIF1-PP1, counteracting the phosphorylation by DDK that triggers replication initiation. We therefore tested whether the MCM hyperphosphorylation characteristic of RIF1-deficient cells could contribute to the overresection phenotype. We found that artificially reducing MCM phosphorylation levels (by using XL-413 to inhibit DDK) did not prevent nascent DNA resection in HU-treated cells lacking RIF1 (Figure S4C), suggesting that the MCM complex is not the RIF1-PP1 dephosphorylation target relevant for nascent DNA protection.

### DNA2-Dependent Resection Compromises Genomic Stability in RIF1-Depleted Cells

**Figure 4:**
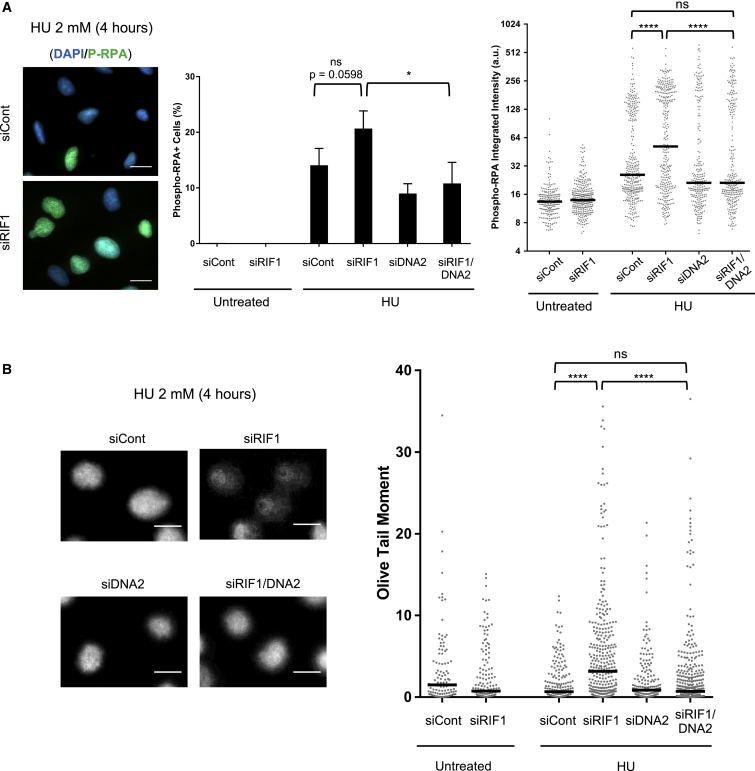
Nucleolytic Processing by DNA2 Underlies DNA Damage Accumulation in the Absence of RIF1 (A) RPA phosphorylation (Ser4/8) was analyzed by immunofluorescence in U2OS cells transfected with the indicated siRNAs. Cells were collected and fixed in unperturbed conditions or after treatment with 2 mM HU for 4 hours. Cells were pulse labeled for 30 min with 5-ethynyl-2ʹ-deoxyuridine (EdU) before collection (untreated samples) or before HU treatment. Left: representative images of phospho-RPA signal. Scale bars, 20 μm. Center: the percentage of cells positive for phospho-RPA (Ser4/8) was quantified for each condition (n = 3 biological replicates). Error bars represent SD. Right: phospho-RPA integrated intensity was measured in EdU^+^ cells. (B) DNA breaks were evaluated by alkaline comet assay in U2OS cells transfected with siRIF1 and/or siDNA2, as indicated. Left: representative images. Scale bars, 5 μm. Right: Olive tail moment values from comet assay, with 2 mM HU treatment for 4 h, as indicated. ns, not significant; ^∗^p ≤ 0.05; ^∗∗∗∗^p ≤ 0.0001.

To investigate the biological consequences of the absence of RIF1 from replication forks challenged by HU, we tested for the appearance of phosphorylated RPA (Ser4/8) that is indicative of DNA damage response activation upon replication stress (Ashley et al., 2014). RIF1 depletion caused an increase in the percentage of cells displaying the phospho-RPA signal after HU treatment (Figure 4A, left and center panels). This increase was significantly suppressed by simultaneous DNA2 depletion, consistent with RPA phosphorylation resulting from the action of DNA2. Similar effects were observed when phospho-RPA intensity was measured specifically in the S phase cell population (Figure 4A, right panel). We next carried out comet assay experiments in alkaline conditions to detect single and double DNA strand breaks. While HU treatment alone did not detectably increase DNA damage, RIF1-depleted HU-treated cells showed a significantly increased tail moment, indicative of an accumulation of broken DNA (Figure 4B). The increased tail moment was greatly reduced when DNA2 was depleted in addition to RIF1, strongly indicating that the DNA2-dependent degradation of the stalled forks in RIF1-deficient cells is responsible for the accumulation of DNA damage. The assessment of breaks was carried out in cells collected immediately after a 4-h treatment with HU, suggesting that the damage accumulation arises directly from replication-associated problems rather than from consequential problems at later cell-cycle stages, such as mitotic abnormalities. We found, however, that depletion of DNA2 was not able to prevent the sensitivity of RIF1-depleted cells to HU (Figure S4D), perhaps because RIF1 has other, DNA2-independent, important roles in enabling the recovery of cells from replication inhibition.

## Discussion

The results described here illuminate the importance of the human RIF1 protein in maintaining the integrity of nascent DNA when replication is challenged. Budding yeast studies suggest that this control by RIF1 is evolutionarily conserved (Hiraga et al., 2018), but provide no information on the mechanism. Here, we have shown that RIF1 must interact with PP1 to prevent the degradation of nascent DNA at stalled forks. We identified the DNA2-WRN nuclease-helicase complex as principally responsible for degradation. We found that RIF1 regulates WRN phosphorylation at a group of sites implicated in controlling its function in resection. WRN may therefore represent an important target for RIF1-PP1 in suppressing unscheduled degradation at blocked forks. Mutating the identified sites to prevent their phosphorylation did not, however, prevent WRN from supporting nascent DNA resection (Figure S3C), indicating that other phosphosites are likely to contribute (perhaps redundantly) to controlling resection by DNA2-WRN. Additional relevant target sites for dephosphorylation by RIF1-PP1 could be on WRN or other proteins, with DNA2 itself being one potential candidate. Little is known about any importance of DNA2 phosphorylation in higher eukaryotes. However, in budding yeast Cdk1-dependent phosphorylation of Dna2 promotes the resection of DSBs (Chen et al., 2011), while in fission yeast, the Cds1 kinase phosphorylates Dna2 on S220 to regulate its association with stalled forks and control its nuclease activity, in turn modulating the formation or stability of reversed forks (Hu et al., 2012). This S220 residue is not conserved in mammals, but human DNA2 may nonetheless be CDK regulated. We did not, however, identify DNA2 as a candidate target of RIF1 in chromatin proteomics analysis (Hiraga et al., 2017).

RIF1 therefore joins a group of proteins, including Abro1, Bod1L, and CtIP, suggested to control the rate of DNA2-mediated resection at stalled replication forks. It remains unclear exactly how these proteins affect DNA2 nuclease activity. Thangavel et al. (2015) showed that after long periods of replication inhibition, DNA2-mediated resection of DNA occurs even in normal cells. We therefore suspect that in the absence of RIF1-PP1, a physiological resection mechanism that normally processes stalled forks is accelerated, rather than an entirely aberrant novel mechanism arising. This suggestion is also consistent with our finding that forks can restart normally following fork blockage in the absence of RIF1 (Figure S1D). Our results are consistent with the requirement for DNA2 in fork restart (Thangavel et al., 2015) since DNA2 was present in our restart experiment.

The function of RIF1 in protecting nascent DNA appears distinct from the BRCA-dependent pathway of fork protection. In BRCA2-defective cells, MRE11 nuclease mediates the degradation of nascent DNA at the stalled forks, but MRE11 is not required for the DNA degradation occurring in the absence of RIF1 (Figures 3B and S2C). At DSBs, RIF1 cooperates with 53BP1 to block BRCA-mediated resection and promote non-homologous end joining (NHEJ) repair. At stalled forks RIF1, however, appears to act independently of 53BP1, since 53BP1-deficient mouse B cells were reported to be proficient in protecting nascent DNA upon genotoxic stress (Ray Chaudhuri et al., 2016).

Therefore, distinct mechanisms seem to protect nascent DNA from degradation upon replication blockage, reflecting the regulation of different nucleases, and many questions remain to be solved about how different pathways intercommunicate to protect nascent DNA at blocked forks and process the structures formed to restart replication. It seems likely that the nuclease activities of MRE11, DNA2, and EXO1 must be separately downregulated by various pathways to prevent nascent DNA degradation. Here, we have identified RIF1-PP1 regulation of the DNA2-WRN complex as an important part of this control network, acting to preserve recently synthesized DNA at stalled forks from DNA2-dependent degradation and limiting the accumulation of DNA damage.

## STAR★Methods

### Key Resources Table

**Table:** 

REAGENT or RESOURCE	SOURCE	IDENTIFIER
**Antibodies**		

Rabbit polyclonal anti-RIF1	Bethyl	Cat# A300-568A; RRID:AB_669806
Rabbit monoclonal anti-phospho-Chk1 (Ser345) [133D3]	Cell Signaling	Cat# 2348; RRID:AB_331212
Rabbit polyclonal anti-DNA2	Abcam	Cat# ab96488; RRID:AB_10677769
Mouse monoclonal anti-MRE11 [12D7]	Abcam	Cat# ab214; RRID:AB_302859
Mouse monoclonal anti-BRCA2 [2B]	Millipore	Cat# OP95; RRID:AB_2067762
Mouse monoclonal anti-CtIP [D-4]	Santa Cruz	Cat# sc-271339; RRID:AB_10608728
Rabbit polyclonal anti-BLM	Abcam	Cat# ab2179; RRID:AB_2290411
Mouse monoclonal anti-WRN (D-6)	Santa Cruz	Cat# sc-376182; RRID:AB_10988219
Mouse monoclonal anti-FLAG M2	Sigma	Cat# F1804; RRID:AB_262044
Rabbit polyclonal anti-RAD51 (H-92)	Santa Cruz	Cat# sc-8349; RRID:AB_2253533
Mouse monoclonal anti-SMARCAL1 (E-12)	Santa Cruz	Cat# sc-376377; RRID:AB_10987841
Rabbit polyclonal anti-phospho MCM2 (S53)	Bethyl	Cat# A300-756A; RRID:AB_669843
Mouse monoclonal anti-RPA32/RPA2 [9H8]	Abcam	Cat# ab2175; RRID:AB_302873
Rabbit polyclonal anti-phospho RPA32 (S4/S8)	Bethyl	Cat# A300-245A; RRID:AB_210547
Rat monoclonal anti-BrdU [BU1/75 (ICR1)]	Abcam	Cat# ab6326; RRID:AB_305426
Mouse monoclonal anti-BrdU	BD Biosciences	Cat# 347580; RRID:AB_400326
Mouse monoclonal anti-ssDNA, clone 16-19	Millipore	Cat# MAB3034; RRID:AB_11212688
Goat anti-Rat IgG (H+L), Alexa Fluor 594	Thermo Fisher	Cat# A-11007; RRID:AB_10561522
Goat anti-Mouse IgG1, Alexa Fluor 488	Thermo Fisher	Cat# A-21121; RRID:AB_2535764
Goat anti-Mouse IgG2a, Alexa Fluor 350	Thermo Fisher	Cat# A-21130; RRID:AB_2535770
Goat anti-Mouse IgG, Cyanine5 conjugated	Thermo Fisher	Cat# A10524; RRID:AB_2534033
Goat anti-Rabbit IgG, Cyanine3 conjugated	Thermo Fisher	Cat# A10520; RRID:AB_2534029

**Chemicals, Peptides, and Recombinant Proteins**		

Hydroxyurea	Sigma	Cat# H8627
Doxycycline hyclate	Sigma	Cat# D9891
Tautomycetin	Tocris	Cat# 2305
Mirin	Sigma	Cat# M9948
NSC-105808 (DNA2 inhibitor)	Gift from Alessandro Vindigni, described in (Kumar et al., 2017)	N/A
XL-413	Gift from Peter Cherepanov	N/A

**Critical Commercial Assays**		

Click-iT Plus EdU Alexa Fluor 647 Imaging Kit	Thermo Fisher	Cat# C10640
OxiSelect Comet Assay Kit (3-Well Slides)	Cell Biolabs	Cat# STA-350

**Experimental Models: Cell Lines**		

Flp-In T-REx 293	Invitrogen	Cat# R78007
HEK293 (GFP)	Hiraga et al., 2017	N/A
HEK293 (GFP-RIF1)	Hiraga et al., 2017	N/A
HEK293 (GFP-RIF1-pp1bs)	Hiraga et al., 2017	N/A
U2OS	Berndt Müller lab	N/A

**Oligonucleotides**		

Control siRNA (Luciferase GL2)	Dharmacon	Cat# D-001100-01
RIF1 siRNA - Human	Dharmacon	Cat# D-027983-02
DNA2 ON-TARGETplus SMARTpool - Human	Dharmacon	Cat# L-026431-01
MRE11 ON-TARGETplus SMARTpool - Human	Dharmacon	Cat# L-009271-00
CtIP ON-TARGETplus SMARTpool - Human	Dharmacon	Cat# L-011376-00
EXO1 ON-TARGETplus SMARTpool - Human	Dharmacon	Cat# L-013120-00
BLM ON-TARGETplus SMARTpool - Human	Dharmacon	Cat# L-007287-00
WRN ON-TARGETplus SMARTpool - Human	Dharmacon	Cat# L-010378-00
WRN siRNA, targeting sequence 5′-GUGCCAUUAAAUAGGGAAAUU-3′	Dharmacon	N/A
RAD51 ON-TARGETplus SMARTpool - Human	Dharmacon	Cat# L-003530-00
SMARCAL1 ON-TARGETplus SMARTpool - Human	Dharmacon	Cat# L-013058-00

**Recombinant DNA**		

pCMV-Flag-WRN-WT	Gift from Pietro Pichierri (Palermo et al., 2016)	N/A
pCMV-Flag-WRN-4A	This paper	N/A

**Software and Algorithms**		

Prism 7	GraphPad	https://www.graphpad.com/scientific-software/prism/
ImageJ	ImageJ Software	https://imagej.nih.gov/ij/
CellProfiler	CellProfiler Software	https://cellprofiler.org/
OpenComet	OpenComet Software	http://www.cometbio.org/
MaxQuant	Max Planck Institute	https://www.maxquant.org/

### Contact for Reagent and Resource Sharing

Further information and requests for resources and reagents should be directed to and will be fulfilled by the Lead Contact, Anne D. Donaldson (a.d.donaldson@abdn.ac.uk).

### Experimental Model and Subject Details

U2OS cells (female) and HEK293-derived cells (female fetus) were cultured in Dulbecco’s modified Eagle’s medium (DMEM), supplemented with 10% fetal bovine serum (FBS) and 1% penicillin-streptomycin, and maintained in an incubator at 37°C and 5% CO_2_. HEK293-derived cell lines containing GFP, GFP-RIF1 or GFP-RIF1-pp1bs constructs were previously generated using the Flp-In T-REx system (Invitrogen) (Hiraga et al., 2017). Tetracycline-free serum was used for the maintenance of the cell lines with the doxycycline-inducible constructs mentioned above.

### Method Details

#### Drug treatments

HU (Sigma) was used at a concentration of 2 mM or 4 mM. Tautomycetin (Tocris) was used at a concentration of 5 μM. DNA2 inhibitor NSC-105808 (Kumar et al., 2017) was added to the cells at a final concentration of 0.3 μM and Mirin (Sigma) was used at a concentration of 50 μM. XL-413 was used at a final concentration of 10 μM.

#### Construction of WRN mutant plasmid

Plasmid pCMV-Flag-WRN-4A was created by replacing a 1.2 kb *Psh*AI-BglII segment of the plasmid pCMV-Flag-WRN-WT (kindly gifted by Pietro Pichierri) with a synthetic DNA fragment containing four S to A substitutions at Ser-1133, 1139, 1140, and 1141. The sequence of the synthetic DNA is available upon request. The DNA sequence was confirmed for the entire Flag-WRN coding sequences, and part of the promoter.

#### siRNA transfection and DNA transfection

Control siRNA against Luciferase (D-001100-01) and siRNA against RIF1 (D-027983-02), DNA2 (L-026431-01), MRE11 (L-009271-00), BRCA2 (L-003462-00), CtIP (L-011376-00), EXO1 (L-013120-00), BLM (L-007287-00), WRN (L-010378-00), WRN 3′UTR custom, RAD51 (L-003530-00) and SMARCAL1 (L-013058-00) were purchased from Dharmacon. For protein knockdown, cells were transfected with the indicated siRNAs at a final concentration of 50 nM (in co-transfections 50 nM of each siRNA was used) using Lipofectamine RNAiMAX reagent (Thermo Fisher). The day after siRNA transfection cells were re-seeded and grown for a further 48 hours, when cells were collected.

For overexpression of Flag-WRN constructs cells were transiently transfected with 2 μg of pCMV-Flag-WRN-WT or pCMV-Flag-WRN-4A using Lipofectamine 3000 Reagent (Thermo Fisher).

#### DNA fiber assay

Cells were pulse-labeled with 50 μM CldU for 20 min, followed by another pulse of 250 μM IdU for 20 min. Then, fork stalling was induced by addition of 2 mM HU for 4 hours. Cells were harvested and DNA fibers prepared as previously described (Mourón et al., 2013). Cells were lysed on a microscope slide with spreading buffer (200 mM Tris pH 7.4, 50 mM EDTA, 0.5% SDS). After 6 min of incubation, the slides were tilted to allow the DNA suspension to run slowly and spread down the slide. Slides were fixed in cold (−20°C) methanol-acetic acid (3:1). DNA was denatured by incubation in 2.5 M HCl at RT for 30 min. Slides were blocked and incubated with the following primary antibodies for 1 hour at RT in humidity chamber (anti-CldU, Abcam ab6326, 1:100; anti-IdU, BD 347580, 1:100; anti-ssDNA, Millipore MAB3034, 1:100). After washes with PBS, the slides were incubated with the following secondary antibodies (anti-rat IgG Alexa Fluor 594, Molecular Probes A-11007; anti-mouse IgG1 Alexa Fluor 488, Molecular Probes A-21121; anti-mouse IgG2a Alexa Fluor 350, Molecular Probes A-21130). Slides were air-dried and mounted with Prolong (Invitrogen). DNA fibers were imaged under a Zeiss Axio Imager and analyzed using ImageJ. CldU and IdU tract lengths were measured in double-labeled forks and the IdU/CldU ratio was used to quantify the degree of nascent DNA resection. Experiments in Figures 1B, 3A, 3C, S1B, S2E, S4A, and S4B show amalgamated results and median from two biological replicates with at least 75 fibers analyzed per condition in each replicate. Experiment in Figures 1C, 3B, S2A–S2D, S3C, and S4C show results and median from one experiment with at least 100 fibers analyzed per condition. Analysis of statistical significance was performed using a Mann-Whitney test (GraphPad Prism).

#### Western blotting and chromatin fractionation

For whole cell protein extraction cells were lysed in SDS buffer (125 mM Tris-HCl pH 6.8, 4% SDS, 20% glycerol) supplemented with protease and phosphatase inhibitors. 50 μg of protein was separated by SDS-PAGE using precast gels (Bio-Rad), transferred to PVDF membranes and probed with the indicated primary antibodies. After incubation with the corresponding HRP-conjugated secondary antibodies, the blots were developed with Clarity ECL reagents (Bio-Rad) and imaged with the ChemiDoc system (Bio-Rad).

For chromatin fractionation, cell pellets were resuspended in CSK buffer (10 mM PIPES-NaOH pH 6.8, 300 mM sucrose, 100 mM NaCl, 3 mM MgCl_2_) supplemented with protease and phosphatase inhibitors. After incubation for 10 min on ice, cells were centrifuged at 2,500 *g* for 3 min and soluble and insoluble fractions separated. The insoluble, chromatin fraction was further resuspended in CSK buffer and incubated 30 min in the presence of benzonase on ice. Samples were then mixed with Laemmli Buffer 2x and incubated for another 30 min at 65°C.

The following primary antibodies were used for immunoblotting: RIF1 (A300-568A, Bethyl Laboratories), phospho-CHK1 (Ser345) (#2348, Cell Signaling), DNA2 (ab96488, Abcam), MRE11 (ab214, Abcam), BRCA2 (OP95, Millipore), CtIP (sc-271339, Santa Cruz Biotechnology), BLM (ab2179, Abcam), WRN (sc-376182, Santa Cruz Biotechnology), FLAG-M2 (F1804, Sigma), RAD51 (sc-8349, Santa Cruz Biotechnology), SMARCAL1 (sc-376377, Santa Cruz Biotechnology), phospho-MCM2 (Ser53) (A300-756A, Bethyl Laboratories).

#### Immunofluorescence

For RPA and phospho-RPA immunostaining, cells were grown on coverslips and pre-extracted on ice with pre-extraction buffer (50 mM NaCl, 300 mM sucrose, 0.5% Triton X-100, 3 mM MgCl_2_, 20 mM HEPES pH 7.9). After fixation with 4% paraformaldehyde, cells were permeabilized by incubating again with pre-extraction buffer for additional 10 min. Coverslips were blocked with 2.5% BSA, 10% Goat Serum in PBS, and incubated with primary antibody at RT for 2 hours, followed by incubation with the secondary antibody for 1 hour. In the case of EdU (5-ethynyl-2′-deoxyuridine) incorporation, cells were pulse-labeled at a concentration of 20 μM for 30 min. EdU detection was performed using the Click-iT Plus EdU Alexa Fluor 647 Imaging Kit from Thermo Fisher, following manufacturer’s instructions. Primary antibodies were anti-RPA32 (ab2175, Abcam) and anti-phospho RPA32 (S4/S8) (A300-245A, Bethyl Laboratories). Secondary antibodies were anti-mouse Cy5 (A10524, Thermo Fisher) and anti-rabbit Cy3 (A10520, Thermo Fisher). Coverslips were finally washed with PBS, stained with DAPI and mounted with Prolong (ThermoFisher), before image acquisition in Zeiss Axio Imager microscope. For phospho-RPA quantification, the percentage of cells with positive signal for Ser4/Ser8 phosphorylation was quantified. For evaluation of fluorescence intensity, integrated intensity was measured in nuclei areas of whole cell population or EdU positive cells. Median values are represented on the scattered dot plots. Images were analyzed using ImageJ and CellProfiler software.

#### Clonogenic assay

For colony survival studies, 24 h after siRNA-mediated protein knockdown, cells were plated at low density in 6 well/plates. One day later, cultures were treated with HU at the indicated times and concentrations. HU-containing media was then replaced with fresh media and cells were kept for 7 days in culture to allow colony formation. Colonies were fixed with 4% paraformaldehyde and stained with 0.02% Crystal Violet. Colonies were counted and the surviving fraction calculated relative to the siControl untreated sample.

#### Alkaline comet assay

Alkaline comet assays were performed according to instructions using OxiSelect Comet Assay Kit (Cell Biolabs). Briefly, harvested cells were mixed with low melting agarose and transferred to an OxiSelect Comet slide. Slides were immersed in lysis buffer for 60 min at 4°C. Lysis buffer was replaced with alkaline solution (300 mM NaOH, pH > 13, 1 mM EDTA) and samples were kept in the dark for 30 min. Slides were transferred to an electrophoresis chamber filled with alkaline solution and electrophoresis was performed for 15 min (1 V/cm). DNA was stained with Vista Green DNA Dye and images were captured by fluorescence microscopy (Zeiss Axio Imager). Comets were scored and tail moment was analyzed using the OpenComet software. The median value is shown on the dot plot.

#### Immunoprecipitation and MS analysis

pCMV-Flag-WRN and plasmids derived from it were transiently transfected to achieve overexpression of WRN constructs. For Flag-WRN immunoprecipitation (IP), cells were collected with a scraper in cold PBS. Cells were lysed in 50 mM Tris-Cl pH 7.5, 150 mM NaCl, 0.5% NP-40, 2 mM MgCl_2_, supplemented with protease/phosphatase inhibitors and benzonase. 1 mg of protein lysate was incubated overnight with anti-Flag M2 antibody (F1804, Sigma) coupled to Dynabeads protein G. After two washes with lysis buffer, beads were further washed with 100 mM ammonium bicarbonate. Immunoprecipitates were processed for on-beads digestion by Trypsin essentially as described (Mohammed et al., 2016). Peptides were then reduced with 10 mM TCEP and alkylated with 15 mM Iodoacetamide. Peptides were analyzed using an Orbitrap Q Exactive Plus mass spectrometer equipped with nano-LC C18 liquid chromatography over 60-min elution gradient. The raw MS datasets were analyzed for label-free quantification by MaxQuant software (version 1.6.2.3). MS1 Intensity of each phosphorylation site was normalized by summed MS1 intensities of WRN in each sample. The normalized phospho/WRN values (expressed as ppm of total WRN) between samples were compared. Since phosphorylations at S1139, S1140, or S1141 could not be unambiguously distinguished, values at these sites were summed.

### Quantification and Statistical Analysis

For column graphs, the mean ± standard deviation (SD) is shown and statistical analysis using Student’s t test was performed. For scatter dot plots (IdU/CldU ratio in fork protection assays, integrated intensity in immunofluorescence assays and tail moment in comet assays), the median value is shown and data were analyzed and statistical significance calculated using Mann-Whitney test for non-parametric distributions. Statistical analysis was performed with GraphPad Prism software (v.7) (ns not significant; ^∗^ p ≤ 0.05; ^∗∗^ p ≤ 0.01; ^∗∗∗^ p ≤ 0.001; ^∗∗∗∗^ p ≤ 0.0001).

## Supplemental Information

Document S1. Figures S1–S4

Document S2. Article plus Supplemental Information
