# Supplementary material for: Human RIF1-Protein Phosphatase 1 Prevents Degradation and Breakage of Nascent DNA on Replication Stalling
Source: Cell Rep. 2019 May 28;27(9):2558–2566.e4. doi: 10.1016/j.celrep.2019.05.002 (PMC6547018; doi:10.1016/j.celrep.2019.05.002)
Supplement: Document S1. Figures S1–S4 [file mmc1.pdf]

Cell Reports, Volume *27*

Supplemental Information

Human RIF1-Protein Phosphatase 1 Prevents Degradation and Breakage of Nascent DNA on Replication Stalling

Javier Garzón, Sebastian Ursich, Massimo Lopes, Shin-ichiro Hiraga, and Anne D. Donaldson

Garzón_Supplemental_Fig_S1

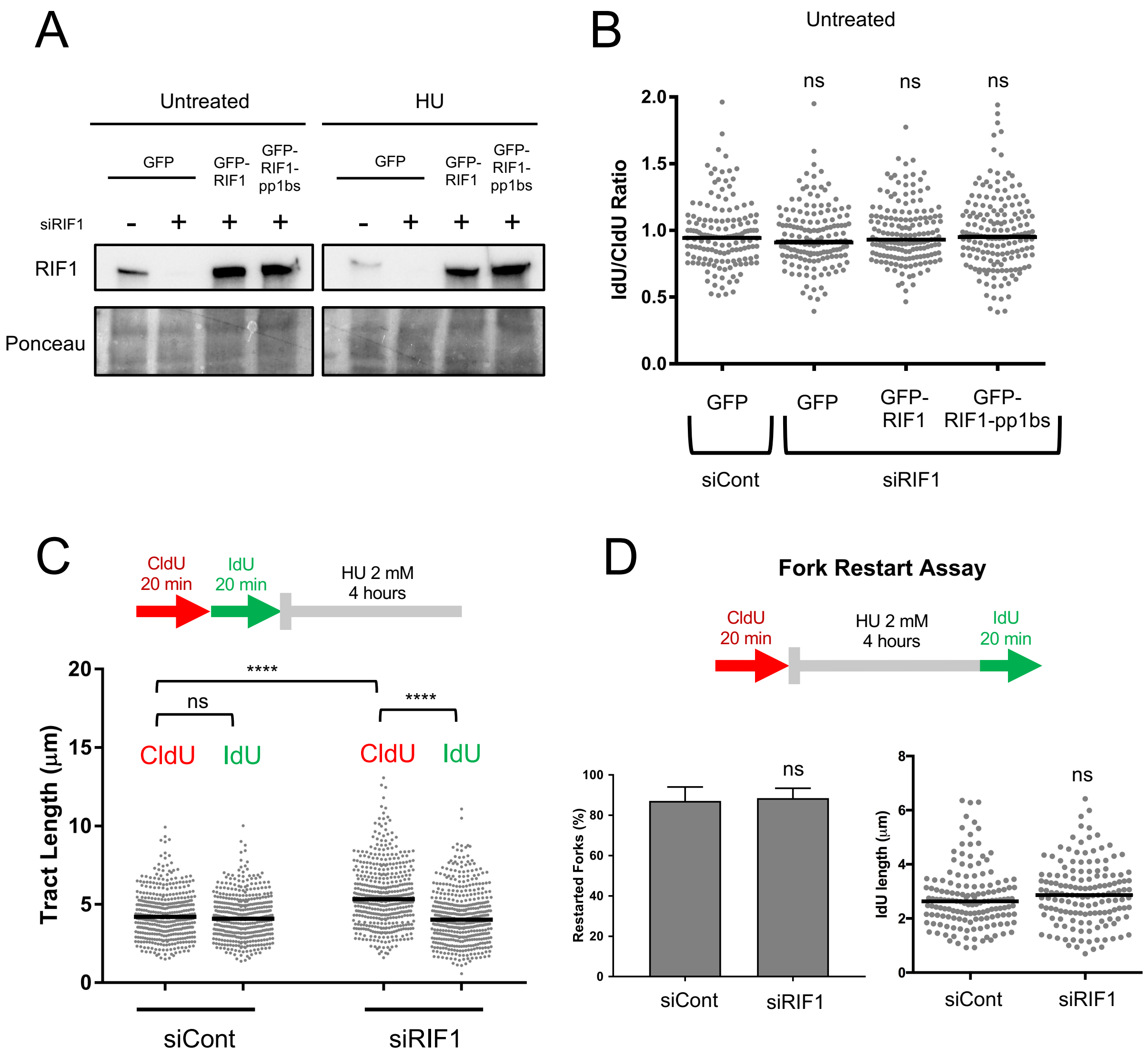

Figure S1 (Related to main Fig. 1). RIF1 is not required for fork restart after HU removal.

(A) Expression of ectopic GFP-RIF1 and GFP-RIF1-pp1bs proteins. Endogenous RIF1 was depleted from HEK293-derived cell lines, then the following day, expression of the stably integrated GFP, GFP-RIF1, GFP-RIF1-pp1bs was induced by addition of doxycycline (DOX). After 2 days cells were treated or not with HU at 2 mM for 4 hours, and RIF1 expression levels assessed by Western blot analysis of whole cell extracts. **(B)** RIF1 does not affect IdU/CldU ratio in untreated HEK293-derived cell lines. HEK293-derived cells were transfected with siCont or siRIF1, and after 24 hours DOX was added to induce expression of GFP, GFP-RIF1, GFP-RIF1-pp1bs as indicated. 2 days after DOX induction the asynchronous cultures were labeled with CldU and IdU (consecutive pulses of 20 min each) and IdU/CldU ratio was calculated in DNA fiber assays. **(C)** CldU and IdU tract length was measured in HEK293-derived cells transfected with siCont or siRIF1 as indicated. More than 400 forks were analysed per condition. **(D)** RIF1 does not affect fork restart. Upper panel, schematic outline of fork restart assay. Cells were first labeled with CldU, followed by HU treatment to induce fork stalling. Upon HU release, IdU was added for 20 min then replication resumption was assessed by fiber assay. Lower panel left, percentage of restarted forks (red-green tracts) out of total number of CldU-labelled fork tracts quantified in the DNA fiber preparation. Error bars represent standard deviation. Lower panel right, IdU tract lengths, measured for replication forks that restarted after HU inhibition.
ns not significant; **** $p \leq 0.0001$

Garzón_Supplemental_Fig_S2

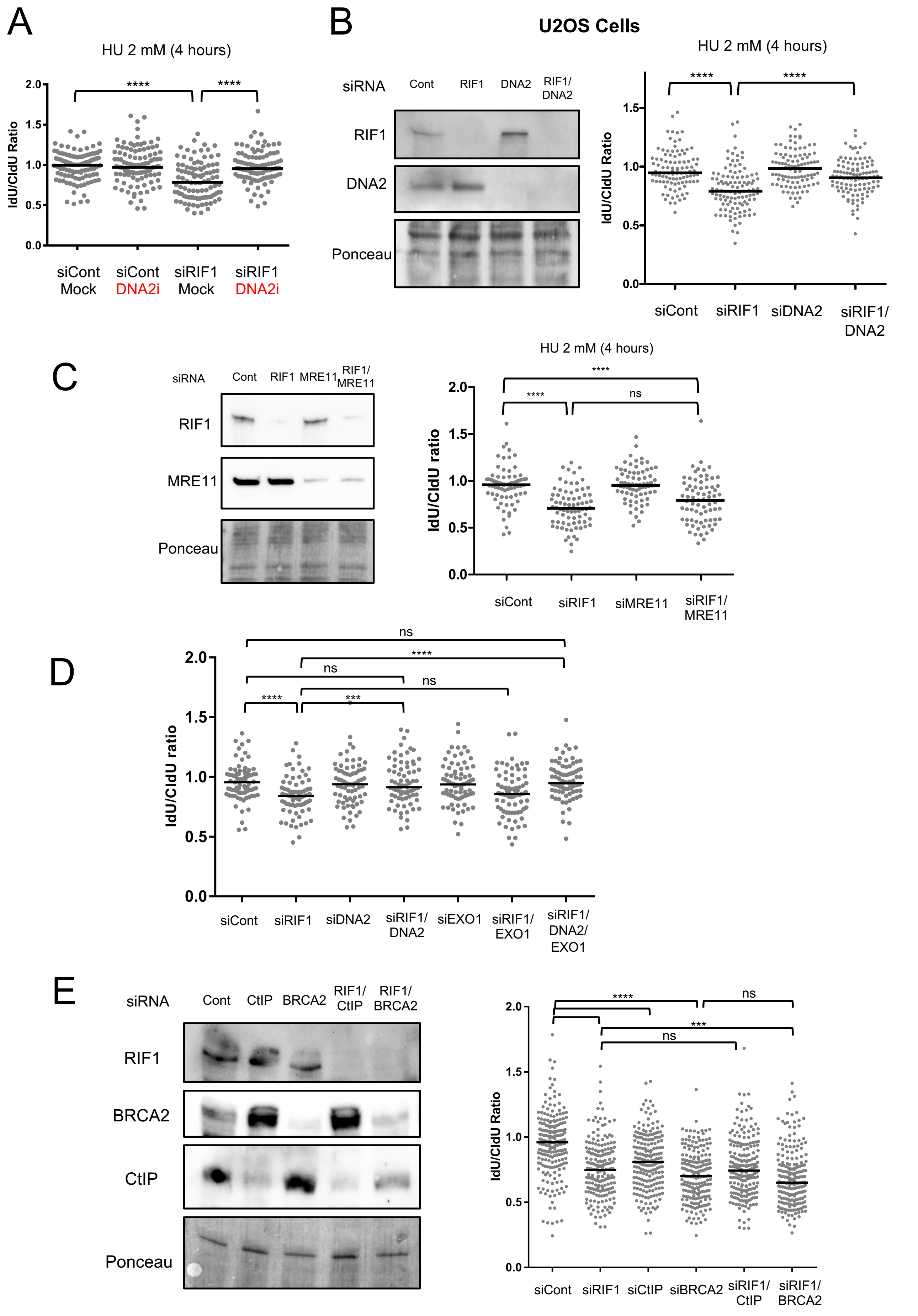

Figure S2 (Related to main Fig. 3). DNA2 depletion/inhibition ameliorates nascent DNA degradation upon RIF1 loss, but removing proteins of the BRCA2-dependent fork protection pathway does not prevent degradation.

(A) DNA2 inhibition prevents DNA degradation. Fork protection assay in cells transfected with siCont or siRIF1 and treated for 24 h with 0.3 μM DNA2 inhibitor NSC-105808, where indicated. **(B)** DNA2 depletion prevents nascent DNA degradation in U2OS cells. Left: RIF1 and DNA2 levels analysed by immunoblotting of U2OS whole cell lysates, after protein knockdown with the indicated siRNAs. Right: Nascent DNA protection after HU treatment, assessed in U2OS cells depleted for RIF1 and/or DNA2. **(C)** RIF1 and MRE11 protein levels in whole cell extracts after transfection with siRIF1, siMRE11, or both, assessed by Western blotting (left panel). Nascent DNA degradation analysed after treatment with 2 mM HU for 4 hours in the indicated conditions (right panel). **(D)** Nascent DNA degradation upon treatment with 2 mM HU for 4 hours in cells depleted for RIF1, DNA2 or EXO1, or co-depleted for RIF1/DNA2, RIF1/EXO1, or RIF1/DNA2/EXO1. **(E)** HEK293 cells were transfected with the indicated siRNAs and analysed for nascent DNA degradation as in previous experiments, following a treatment with 2 mM HU for 4 hours. Left: protein levels were evaluated by Western blotting after siRNA transfection. Right: IdU/CldU ratio for the indicated conditions after treatment with HU.

ns not significant; *** $p < 0.001$; **** $p \leq 0.0001$

Garzón_Supplemental_Fig_S3

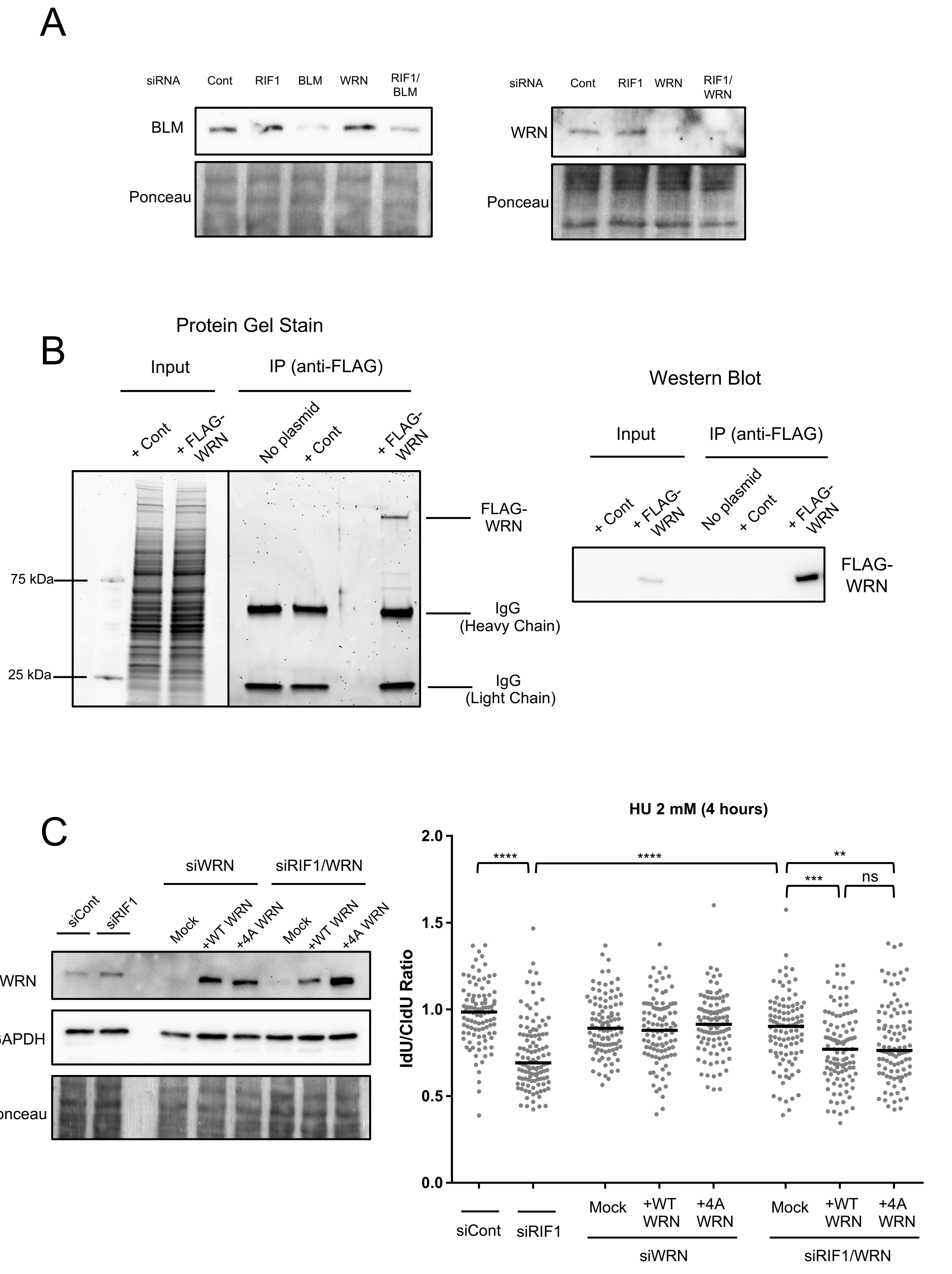

Figure S3 (Related to main Fig. 3). WRN contributes to nascent DNA degradation and is differentially phosphorylated in the absence of RIF1

(A) BLM and WRN helicase protein levels analysed by immunoblotting after siRNA transfection in HEK293 cells. **(B)** Flag-WRN immunoprecipitation. HEK293 cells were transiently transfected with a Flag-tagged WRN plasmid or with a Control plasmid expressing GFP. Cells were treated or not with HU 2 mM for 4 hours. Samples were immunoprecipitated with an anti-Flag antibody. Left: input and IP samples were run in a SDS-PAGE gel and stained for total protein with Coomassie Fluor Orange. The two sides of the image are from the same gel (input lanes shown at a low exposure and IP lanes at a longer exposure). Gel shown corresponds to untreated samples. Right: a portion of the same input and IP extracts was used for Western blotting and probed against anti-Flag antibody to verify IP efficiency. **(C)** HEK293 cells were transfected or co-transfected with siCont, siRIF1, siWRN or siRIF1/WRN. 24 hours later, WRN constructs encoding wild-type WRN (WT WRN) or a mutated version non-phosphorylatable at Ser residues 1133/1139/1140/1141 (4A WRN) were transfected, or else cells were Mock-transfected using no plasmid. Note that these WRN constructs were resistant to siRNA, as the oligonucleotide used to silence expression of endogenous protein was specifically designed to target a sequence within the 3'UTR region of WRN that is absent from the Flag-WRN transient expression plasmids. 3 days after siRNA transfection (2 days after plasmid transfection) fork stalling was induced with HU and nascent DNA degradation evaluated as in previous figures. Left: immunoblotting of whole cell extracts showing WRN protein levels after depletion of endogenous protein and expression of the indicated WRN-constructs. Right: fork protection assay after treatment with HU 2 mM for 4 hours.
ns not significant; ** $p< 0.01$; *** $p< 0.001$; **** $p \leq 0.0001$

Garzón_Supplemental_Fig_S4

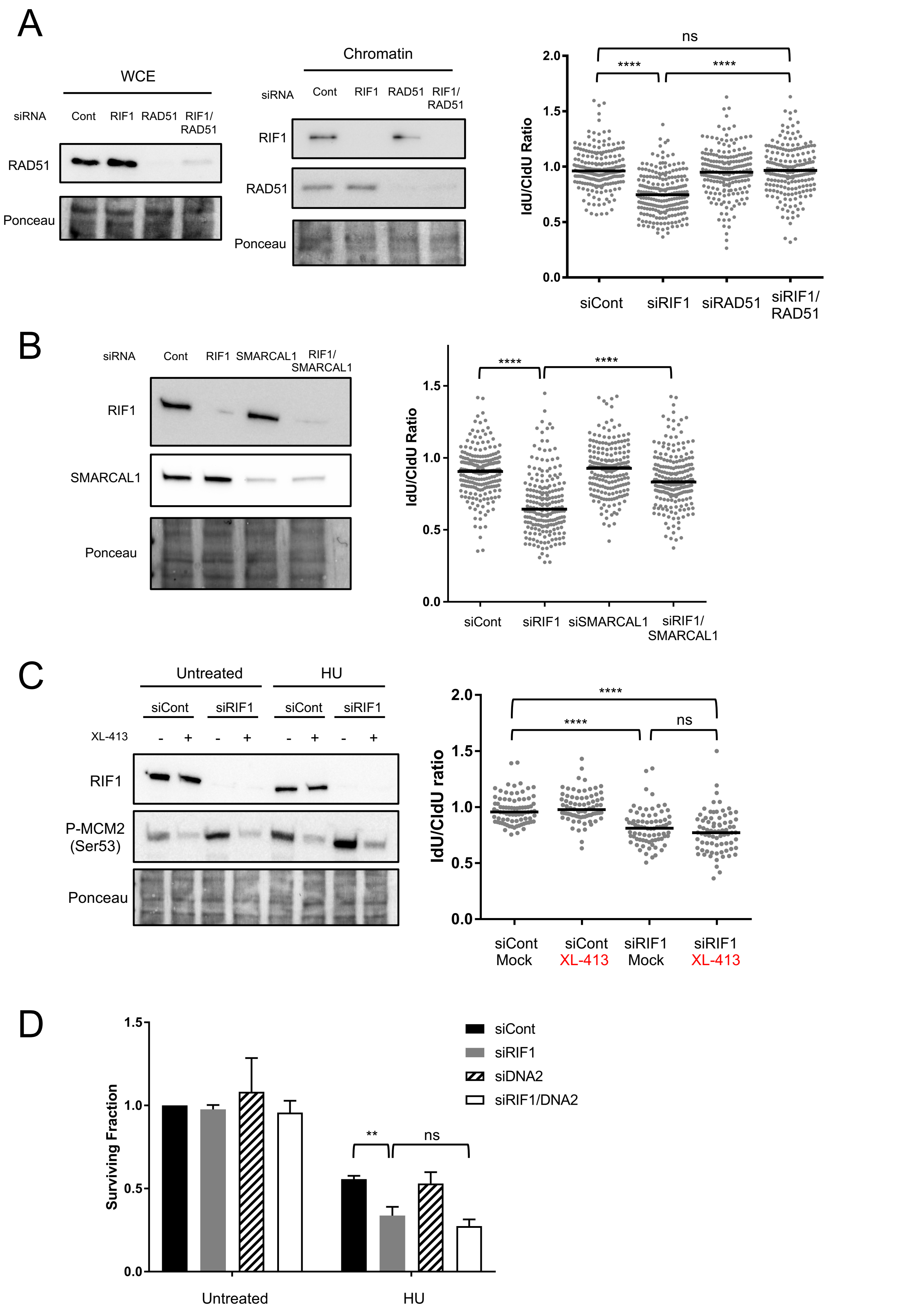

Figure S4 (Related to main Fig. 4). Depletion of fork reversal factors prevents nascent DNA over-resection in cells lacking RIF1; nascent DNA protection is not mediated by MCM dephosphorylation; and RIF1 hypersensitivity to HU is not due to DNA2-dependent degradation.

(A) Left: Western blot analysis of HEK293 whole cell extracts and chromatin fractions after siRIF1 and/or siRAD51 transfection. Right: Nascent DNA degradation analysis by DNA fiber assays after HU treatment (2 mM for 4 hours) in cells depleted from RIF1 and/or RAD51. (**B**) Left: RIF1 and SMARCAL1 protein levels assessed by immunoblotting after transfection of HEK293 cells with the indicated siRNAs. Right: Fork protection assay in cells transfected with the indicated siRNAs and treated with HU 2 mM for 4 hours. (**C**) Left panel, immunoblot of chromatin-enriched fractions from HEK293 cells transfected with siCont or siRIF1, after DDK inhibition with XL-413. Membrane was probed with phospho-specific antibody against phospho-MCM2 (Ser53) to confirm the expected changes in MCM phosphorylation caused by DDK inhibition. Right panel, fork protection analysis from DNA fiber samples after DDK inhibition with XL-413. 10 µM XL-413 was added 30 min before HU treatment. (**D**) Clonogenic assay. HEK293 cells were transfected with the indicated siRNAs. Cells were treated (or not) with HU 4 mM for 8 hours. Drug was washed off and cells seeded at low density to assess viability. Surviving colonies were counted 7 days after treatment (n = 3 independent experiments). Error bars represent standard deviation. ns, not significant; ** $p \leq 0.01$; **** $p \leq 0.0001$
